# Dietary Super-Doses of Cholecalciferol Fed to Aged Laying Hens Illustrates Limitation of 24,25-Dihydroxycholecalciferol Conversion

**DOI:** 10.1016/j.cdnut.2024.102156

**Published:** 2024-04-09

**Authors:** Matthew F Warren, Pete M Pitman, Dellila D Hodgson, Nicholas C Thompson, Kimberly A Livingston

**Affiliations:** 1 Prestage Department of Poultry Science, North Carolina State University, Raleigh, NC, United States; 2 Department of Biological Sciences, North Carolina State University, Raleigh, NC, United States; 3 Department of Animal Science, North Carolina State University, Raleigh, NC, United States

**Keywords:** aged laying hen, chicken, dietary vitamin D_3_, 25-hydroxycholecalciferol, 24,25-dihydroxycholecalciferol, egg, egg yolk vitamin D_3_, plasma vitamin D_3_, super-dose

## Abstract

**Background:**

Older humans taking high concentrations of vitamin D_3_ supplementation for a prolonged time may be at risk of vitamin D toxicity. It is unclear how dietary super-doses (10,000 times greater than the requirement) can affect vitamin D_3_ status in aged animals. Aged laying hens could be a model to compare vitamin D_3_ supplementation effects with women in peri- or postmenopausal stages of life.

**Objectives:**

We investigated the dietary super-dose impacts of cholecalciferol (vitamin D_3_) on vitamin D_3_ status in aged laying hens in production.

**Methods:**

Forty-eight 68-wk-old Hy-Line Brown laying hens were individually housed in cages with 8 hens per dietary treatment for 11 wk. Hens were randomly assigned to 1 of 6 treatment groups of dietary vitamin D_3_ supplementation and consumed *ad libitum*. Supplementation concentrations were 400, 800, 7400, 14,000, 20,000, and 36,000 IU D_3_/kg of feed. At the end of the study, all hens were sacrificed, and tissue samples and feces were collected. Plasma and egg yolk vitamin D_3_ metabolites, calcium and phosphorus composition of eggshells, ileal digesta, and feces were measured. Duodenal, ileal, liver, and kidney gene expression levels were also measured.

**Results:**

We observed that increasing dietary vitamin D_3_ increased plasma vitamin D_3_ and egg yolk vitamin D_3_ (*P* < 0.0001 for both sites). We also observed an increase in plasma 24,25-dihydroxycholecalciferol as dietary vitamin D_3_ concentrations increased (*P* < 0.0001). The plasma 25-hydroxycholecalciferol:24,25-dihydroxycholecalciferol ratio exhibited an asymptotic relationship starting at the 14,000 IU/kg D_3_ treatment.

**Conclusions:**

Dietary super-doses of vitamin D_3_ led to greater plasma and egg yolk vitamin D_3_ concentrations, which shows that aged laying hens can deposit excess vitamin D_3_ in egg yolk. We suggest future research should explore how 24-hydroxylation mechanisms are affected by vitamin D_3_ supplementation. Further understanding of 24-hydroxylation can help ascertain ways to reduce the risk of vitamin D toxicity.

## Introduction

Older humans taking extremely high concentrations of vitamin D_3_ supplementation for a prolonged time may be at risk of vitamin D toxicity [1,2]. People tend to take vitamin D_3_ supplements to increase or maintain vitamin D_3_ concentrations [3]. Also, older women take vitamin D_3_ supplements to manage the hormonal effects of menopause on bone resorption [[4], [5], [6]]. Although vitamin D toxicity is uncommon, vitamin D_3_ supplements and overfortified foods are the only known means of reaching intoxication levels [1,7]. Vitamin D_3_ supplementation can be administered through multiple means with the common routes being oral dose supplementation or dietary supplementation.

An important consideration is whether extremely high supplementation of vitamin D_3_ concentrations over an extended period of time would cause vitamin D toxicity. Laying hens, chickens that have high egg-laying production, have been used to explore questions involving vitamin D_3_ supplementation [[8], [11]]. Signs of vitamin D toxicity in egg-laying chickens are reduction of food consumption, lower egg production, and reduced growth in younger chickens [10,12]. Laying hens fed a diet containing 68,348 IU of D_3_/kg of feed over a 48-wk period had reduced body weight and egg production, which is suggestive of vitamin D toxicity [10]. Considering older humans are likely to take vitamin D_3_ supplements, further exploration of dietary vitamin D_3_ supplementation effects in older hens is necessary. Characterizing how high concentrations of dietary vitamin D_3_ affect vitamin D metabolism in older hens may help to better understand how overfortified foods can potentially affect vitamin D metabolism in older animals and humans.

Dietary vitamin D_3_ supplementation is important for laying hens in production because their bone health is physiologically taxed from egg production [13,14]. Laying hens in commercial farms are fed diets with supplemental vitamin D_3_ beyond the National Research Council requirements [15,16]. This ensures the hens can lay eggs and maintain adequate calcium (Ca) absorption for eggshell formation and, importantly, bone mineralization [11]. There are a few studies that investigated how very high concentrations of dietary vitamin D_3_ supplementation affected laying hen production and the metabolic implications pertaining to vitamin D_3_ status [9,10,17]. Altogether, the aforementioned studies illustrate dietary vitamin D_3_ supplementation results in vitamin D_3_-enriched eggs which may be a way to improve vitamin D_3_ intake for humans. Further understanding of how very high concentrations of dietary vitamin D_3_ supplementation affect circulating vitamin D_3_ metabolite concentrations in aged laying hens is relevant to the poultry producers interested in extending the production life of laying hens. Also, understanding the impacts of very high dietary vitamin D_3_ supplementation in aged laying hens has implications with older women and their vitamin D_3_ intake from food fortification.

Our study examined dietary vitamin D_3_ super-dose effects on plasma and egg yolk vitamin D_3_ metabolites and relative gene expression of vitamin D-related genes in aged laying hens in production. We define “super-dose” as treatment doses >10,000 times greater than requirement. We fed hens diets containing 400, 800, 7400, 14,000, 20,000, and 36,000 IU D_3_/kg of feed to ascertain vitamin D_3_ supplementation impacts. Hens consuming diets with vitamin D_3_ >7400 IU D_3_/kg were expected to have increased plasma 24,25-dihydroxycholecalciferol [24,25-(OH)_2_-D_3_] because 24,25-(OH)_2_-D_3_ is an inactive form of vitamin D_3_ and would suggest that the hens reached vitamin D_3_ saturation [[18], [19], [20]]. Hens consuming super-doses of vitamin D_3_ should also lay eggs with increased vitamin D_3_ content because they would deposit excess vitamin D_3_ into the egg yolk [9].

## Methods

### Animal husbandry

**FIGURE 1:**
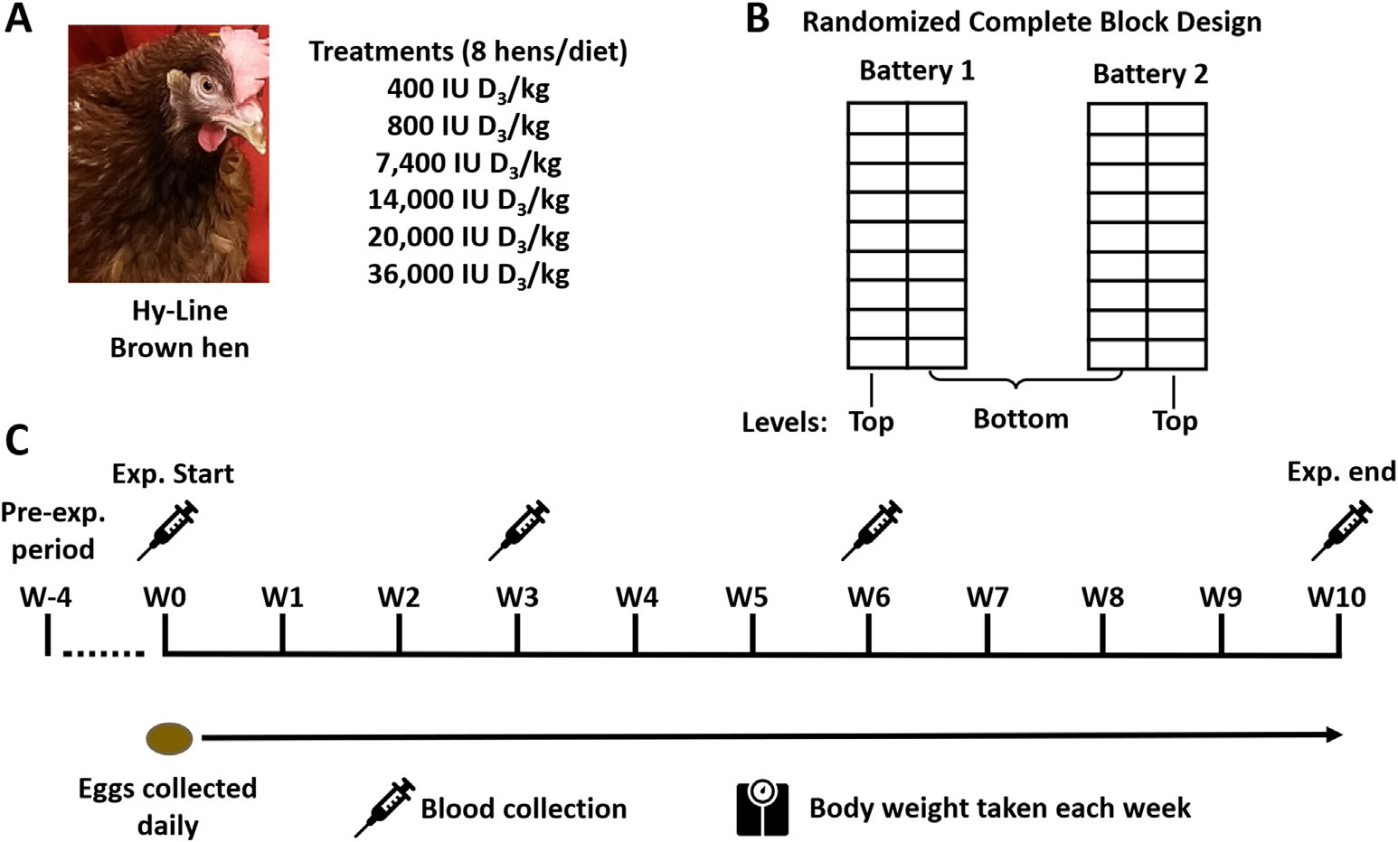
Study design. (A) Forty-eight 68-wk Hy-Line Brown hens were used in the study and were randomly assorted into 1 of 6 treatment groups and fed the diet with the corresponding dietary vitamin D_3_ supplementation concentration. (B) Hens were individually housed in cages of a 2-leveled battery cage in a randomly assigned complete block design (*n* = 8/diet). (C) The experimental timeline in which hens were fed the same basal diet (400 IU D_3_/kg) for 4 wk as a washout period (W-4). Hens were started on the experimental diets at week 0 (W0), eggs were collected daily, and weekly body weight was taken until the end of the study. On weeks 0, 3, 6, and 10, blood was collected from the brachial (wing) vein from all hens to measure ionized blood calcium using an i-STAT blood analyzer. Blood was centrifuged down, and plasma was collected to measure vitamin D_3_ metabolite concentrations. The study ended on week 10, and all hens were sacrificed, and sample tissues were collected.

The hens used in our study were from North Carolina State University’s maintained poultry flock. Forty-eight 68-wk-old Hy-Line Brown laying hens were housed at North Carolina State University, Raleigh, NC, and fed experimental diets for 11 wk (Figure 1). Hens were individually housed in cages between 2 2-level (top level and bottom level) battery cages with 8 hens per treatment. Each hen was individually fed via their own trough feeder with side barriers to reduce cross-feeding between hens and randomly assigned to a treatment group. The experimental design was a randomized complete block design with 6 levels of dietary vitamin D_3_ supplementation blocked by cage level. Vitamin D_3_ supplementation concentrations were formulated to be 250, 500, 1500, 15,000, 30,000, and 60,000 IU D_3_/kg of feed, but the analyzed vitamin D_3_ concentrations in the feed were 400, 800, 7400, 14,000, 20,000, and 36,000 IU D_3_/kg of feed (Table 1 and Supplementary Table 1). The source of vitamin D_3_ used in the study was the crystalline vitamin D_3_ from Alfa Aesar. We refer to the 6 different analyzed vitamin D_3_ concentrations as the named treatment groups for our study. In our study, the 400 and 800 IU/kg vitamin D_3_ treatments were formulated to meet the National Research Council [16] requirements for laying hens. The 7400, 14,000, 20,000, and 36,000 IU/kg D_3_ treatments were dietary super-dose treatments for D_3_ intake. Prior to the start of the experiment, all hens were fed the same diet (400 IU D_3_/kg of feed) for 1 mo as a washout period. Hens were fed the diet and water *ad libitum*. North Carolina State University’s Institutional Animal Care and Use Committee approved all methods for this study, protocol ID number: 18-093-A.

**TABLE 1:** Ingredient composition of the experimental basal diet

Ingredient name	%
Corn	63.40
Soybean meal, 46% crude protein	20.60
Calcium carbonate	9.20
Poultry fat	2.33
Dicalcium phosphate^1^	1.95
L-lysine-hydrochloride, 78.8%	1.01
Sodium bicarbonate	0.57
Vitamin premix^2^	0.25
Mineral premix^3^	0.20
Choline chloride, 60% choline	0.20
DL-Methionine, 99%	0.14
Salt	0.10
Selenium premix^4^	0.05

1 Dicalcium phosphate contains 19.79% calcium, 17.91% phosphorus, and 17.73% available phosphorus.

2 Provided as milligrams per kilogram of diet: 125 mg ethoxyquin; 25 mg niacinamide;10 mg calcium pantothenate; 6.7 mg DL-α-tocopherol; 3.6 mg riboflavin; 3 mg pyridoxine hydrochloride; 1.8 mg thiamine hydrochloride; 0.55 mg folic acid; 0.55 mg menadione sodium bisulfite; 0.516 mg retinol acetate; 0.15 mg biotin; 0.01 mg cyanocobalamin.

3 Trace minerals provided per kg of premix: 60 g manganese sulfate, 60 g zinc sulfate, 40 g iron sulfate, 5 g copper sulfate, 1.25 g calcium iodate.

4 Selenium premix provided selenium at 0.3 mg/kg of diet.

### Sample Collection

Egg collection started 24 h after the hens were started on the experimental diets. Eggs were collected every morning and stored at 7°C for egg quality analyses. Shell strength and elasticity were measured using methods described by Redhead et al. [21], and shell thickness was also measured using calipers. Egg quality measurements were done by selecting 2 eggs at random per week for each replicate. Starting on Mondays, the first egg laid for the week by each hen was selected for egg yolk collection. Eggs were cracked open in a dim-lighted room to reduce the photodegradative impacts of light on vitamin D_3_ in the yolk. The egg yolk was separated from albumen and placed in a small plastic container wrapped in aluminum foil and stored at 4°C for a year until they were freeze-dried using a freeze-dryer (FreeZone 6 Liter Benchtop Freeze Dry System; Labconco). On weeks 0, 3, 6, and 10, blood was collected from the brachial wing vein from all hens to measure ionized blood Ca using an i-STAT blood analyzer (Abaxis) using CG8+ cartridges (Abaxis). The remaining blood was centrifuged down to collect plasma, which was stored at –80°C. All hens were sacrificed by cervical dislocation, and tissue samples were collected from 43 hens (minimum of 7 hens per treatment) due to time constraints. The duodenum, ileum, liver, and kidney were collected and stored in RNAlater at –20°C until RNA extractions were performed. The feces and ileal digesta were collected immediately after the hens were sacrificed, along with the humerus and tibia bones. The ileal digesta, feces, humerus, and tibia were collected for measuring Ca and phosphorus (P) composition.

### Ca and P content of various sites

Eggshells from weeks 0, 3, 4, 6, and 9 were washed with warm water to help with removing the shell membrane by hand and dried for 48 h at room temperature. Dried eggshells were preweighed and further dried at 68°C for 72 h using a dry oven (Blue M) and weighed again. Eggshells were crushed into fine powder and subjected to acid digestion to measure the Ca and P composition of eggshells. Feces and ileal digesta were also subjected to the same steps as eggshells. Dried samples were weighed and then placed in a muffle furnace at 500°C overnight to ash samples. The ashed samples were processed by North Carolina State University’s Environmental and Agricultural Testing Service laboratory. Ashed samples were dehydrated in 2 mL of distilled water and 4 mL of 6 N hydrochloric acid. The resulting sample was mixed and heated to warm to the touch. The heated solution was poured into a volumetric flask, and deionized water was added to have a working solution of 50 mL. The flask was inverted 12 times to mix, and the resulting solution was filtered using #40 filter paper into 15 mL centrifuge tubes for analysis. Ca and P were measured by inductively coupled plasma optical emission spectrometry.

The humerus and tibia were wrapped in petroleum ether-moistened cheesecloth and placed in a desiccator for 72 h to extract fat and moisture from the bones. Fat-extracted bones were preweighed and dried for 24 h at 100°C to evaporate petroleum ether residues. Fat- and moisture-free bones were weighed and ashed using the same methods as eggshell, feces, and ileal digesta for Ca and P composition and measured by inductively coupled plasma optical emission spectrometry.

### RNA extraction and qPCR

Total RNA was extracted from the duodenum, ileum, liver, and kidney using Qiagen’s RNeasy Mini Kit. The extracted RNA was diluted and normalized to ∼200 ng/μL for the liver and 60 ng/μL for the duodenum, ileum, and kidney. The tissues’ RNA was reverse transcribed to cDNA using Applied Biosystems’ High-Capacity cDNA Reverse Transcription Kit (ThermoFisher Scientific) and their recommended steps to make a 20 μL working solution. The cycling procedure for reverse transcription started at 25°C for 10 min, 37°C for 120 min, 85°C for 5 min, then held at 5°C indefinitely until storage or use.

Genes amplified for qPCR were vitamin D receptor (VDR), 1α-hydroxylase (CYP27C1), 24-hydroxylase (CYP24A1), and glyceraldehyde 3-phosphate dehydrogenase (GAPDH) as the housekeeping gene (Table 2). qPCR was conducted using PowerUP SYBR Green Master Mix (Life Technologies) following the manufacturer’s protocol and using Applied Biosystems StepOnePlus Real-Time PCR System. The cycling procedure started at 95°C for 10 min, then 40 cycles at 95°C for 15 s for denaturing and 15 s at 60°C for annealing. All samples were analyzed in triplicates.

**TABLE 2:** Primer sequences for qPCR

Gene	Orientation	Primer sequence (5’ – 3’)	Size (bp)	Accession #
VDR	Forward	TGCCTCCAGTCTGGCATCTC	297	NM_205098.1
	Reverse	GGTGATTTTGCAGTCCCCGT		
CYP27C1	Forward	ATGATTGGCGTCCCCTTCAG	177	XM_422077.4^1^
	Reverse	TCCACGCTTTCACTCACACA		
CYP24A1	Forward	AAACCCTGGAAAGCCTATCG	133	NM_204979.1^2^
	Reverse	CCAGTTTCACCACCTCCTTG		
GAPDH	Forward	TGTTGTTGACCTGACCTGCC	291	NM_204305.1
	Reverse	CTGGCTCACTCCTTGGATGC		

CYP24A1, 24-hydroxylase; CYP27C1, 1α-hydroxylase; GAPDH, glyceraldehyde 3-phosphate dehydrogenase; qPCR, quantitative polymerase chain reaction; VDR, vitamin D receptor; NCBI, National Center for Biotechnology Information.

1 The NCBI record for CYP27C1 was removed due to standard genome annotation processing. However, in our study, the CYP27C1 primers were made and used in 2019 when the record was available.

2 The NCBI record for CYP24A1 was removed because of insufficient support for the transcript and protein. In our study, the CYP24A1 primers were made and used in 2019 when the record was available.

### Vitamin D_3_ metabolites

Plasma from the week 10 timepoint from hens from the 400, 800, 14,000, and 36,000 IU D_3_/kg groups were respectively pooled (*n* = 4/treatment) and sent to Heartland Assays for measuring vitamin D_3_, 25-hydroxycholecalciferol (25-OH-D_3_), and 24,25-(OH)_2_-D_3,_ inactive form of vitamin D_3_, using LC-MS/MS. Freeze-dried egg yolk from week 10 timepoint from the same hens were pooled (15 g/sample) like plasma, but only samples from 400, 14,000, and 36,000 IU D_3_/kg groups (*n* = 4/treatment) were analyzed for vitamin D_3_ and 25-OH-D_3_ from Heartland Assays.

### Statistical analysis

We conducted statistical analyses using general linear models using SAS 9.4 for all statistical tests, and the Tukey-Kramer test was used for multiple comparisons of differences between dietary treatments. We utilized repeated measures to account for the temporal effects of weekly body weight, feed intake, egg production, eggshell quality, and ionized blood Ca. Dietary vitamin D_3_ concentration was the independent variable for Ca and P composition, plasma and egg yolk vitamin D_3_ metabolite concentrations, and gene expression data. Plasma vitamin D_3_ was below the detection limit (<0.5 ng/mL) for the 400 and 800 IU D_3_/kg treatments, so those samples were set to 0.4 as an arbitrary value for statistics to account for model building. All vitamin D_3_ metabolite data exhibited heteroscedasticity and were transformed using the natural logarithm function. Transformed data exhibited linear and homoscedastic relationships and were used for statistics. We did not observe a cage-level effect in any analysis, so the blocking variable was omitted from all statistical tests. All mRNA relative expressions were normalized using 2^-CΔΔT^ with GAPDH as the housekeeping gene. Statistical significance was established at *P* < 0.05.

## Results

### Hens’ production performance was not influenced by dietary vitamin D_3_

To determine if dietary super-doses of vitamin D_3_ affected the hens’ production value, we measured the hens’ weekly body weights and egg production. Dietary super-doses of vitamin D_3_ did not affect the body weight of these laying hens (*P* = 0.08; Supplementary Table 2), but there was an interaction between dietary vitamin D_3_ concentration over time on feed intake (*P* < 0.0001). However, there was constant feed wastage throughout the study so this effect could be inflated. A dietary trend was observed for egg production for the entire study duration (*P* = 0.07; Supplementary Table 3). Eggshell strength and eggshell thickness were not affected by dietary vitamin D_3_ concentrations (*P* = 0.19, *P* = 0.72, respectively; Supplementary Table 3). There was a trending interaction between dietary vitamin D_3_ concentrations over time in which there was a decrease in eggshell elasticity (*P* = 0.07).

### Ionized blood Ca is affected by dietary vitamin D_3_ concentrations

We also examined if ionized blood Ca in hens was influenced by dietary vitamin D_3_ concentrations throughout the study. There was no temporal effect on ionized blood Ca (*P* = 0.65). With week 0 considered as a covariate, there was a dietary effect on ionized blood Ca (*P* = 0.002, Supplementary Table 4). It is not known why there is no clear trend in ionized blood Ca relative to the dietary treatment, but one possibility could be related to when the hen laid an egg prior to the blood collection, which may influence circulating Ca concentrations.

### Fecal Ca is affected by dietary vitamin D_3_ concentrations, but not ileal digesta or eggshell Ca or P

We had the eggshell, ileal digesta, and fecal Ca and P measured to determine if dietary vitamin D_3_ fed to our hens would reduce the excretion or loss of Ca and P. There was no dietary effect on eggshell Ca and P (*P* = 0.64 for both) and ileal digesta Ca or P (*P* = 0.74 and 0.09, respectively). There was a dietary effect with fecal Ca with hens fed 14,000 IU/kg D_3_ diet had 10.6% ± 0.8% Ca by weight in their feces, whereas all other treatments were 8.0–9.0%, with the exception of 36,000 IU/kg D_3_ fed hens which had 7.36% ± 0.43% fecal Ca (*P* = 0.03, Supplementary Table 5). There was no dietary effect on fecal P (*P* = 0.76).

### Humerus is more Ca and P dense than tibia

We assessed if dietary vitamin D_3_ would improve bone Ca and P in hens. However, no dietary effects of vitamin D_3_ supplementation were observed on bone Ca or P (*P* = 0.79 and 0.63, respectively). Humerus bones have a higher percentage by weight, Ca and P, than tibia bones (*P* = 0.020 and 0.015, respectively; Supplementary Figure 1).

### Dietary super-dosage concentrations of vitamin D_3_ increased plasma and egg yolk vitamin D_3_ metabolites

**FIGURE 2:**
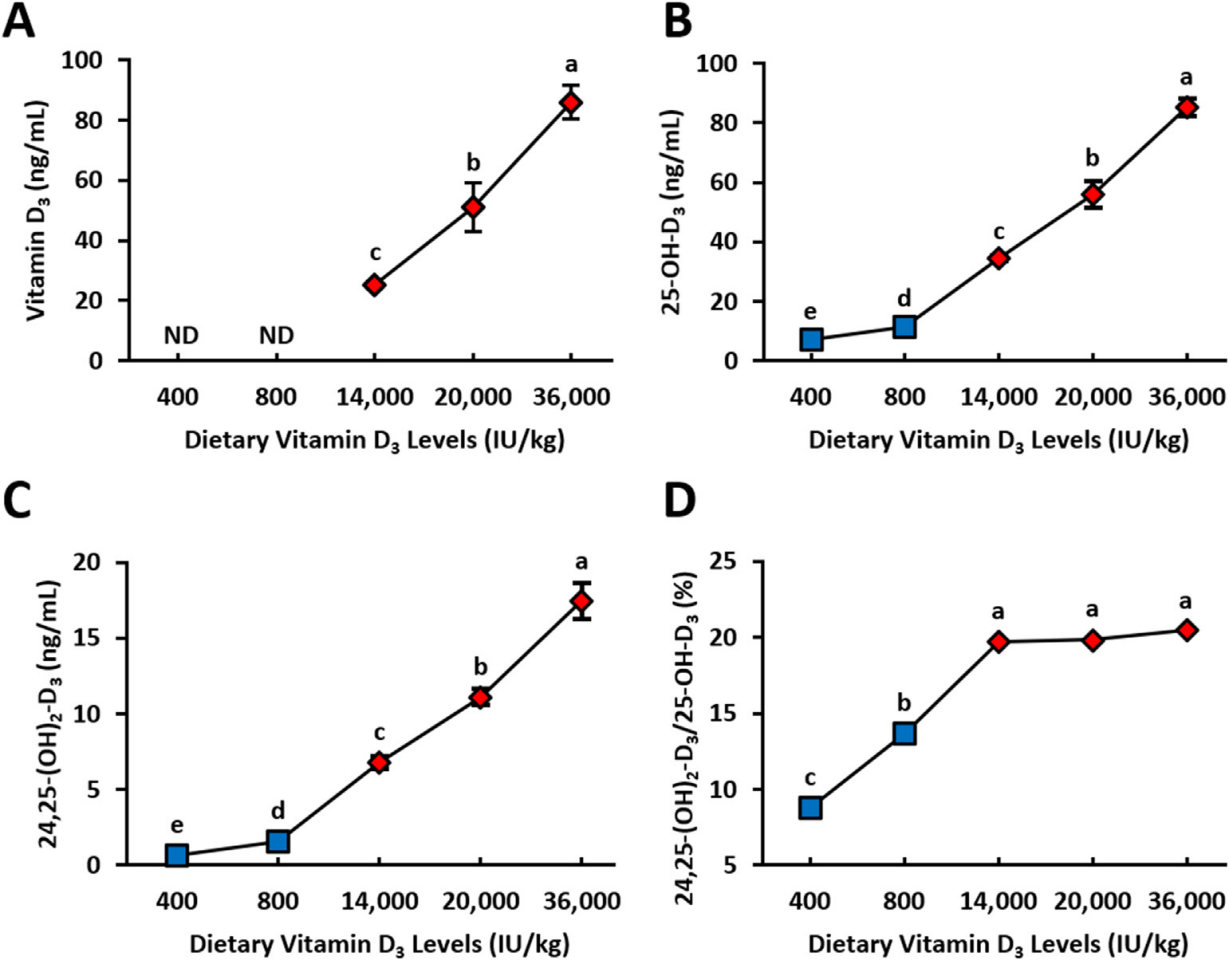
Vitamin D_3_ metabolite plasma concentrations of 78-wk Hy-Line Brown laying hens fed different concentrations of dietary vitamin D_3_. (A) Cholecalciferol [vitamin D_3_; 400 and 800 IU treatment concentrations were below limit of detection and were not determined (ND)] (B) 25-hydroxycholecalciferol (25-OH-D_3_) (C) 24,25-dihydroxycholecalciferol [24,25-(OH)_2_-D_3_] (D) ratio of 24,25-(OH)_2_-D_3_/25-OH-D_3_ presented as a percentage. Blue squares denote standard NRC range vitamin D_3_ concentrations (400 and 800 IU D_3_/kg) in diet (*n* = 4), and red diamonds denote super-dose concentrations of vitamin D_3_ (14,000, 20,000, and 36,000 IU D_3_/kg) in diet (*n* = 4). Samples were reported as means ± SEM. Samples with common letters were not significantly different from each other (general linear models, *P* < 0.0001). NRC, National Research Council; SEM, standard error of the mean.

We had plasma and egg yolk vitamin D_3_ metabolites measured by LC-MS/MS to determine if dietary vitamin D_3_ affected plasma and egg yolk concentrations. There was a significant increase in plasma concentration of vitamin D_3_, 25-OH-D_3_, and 24,25-(OH)_2_-D_3_ of hens fed dietary super-dose concentrations of vitamin D_3_ (**D**_**3**_: *P* = 0.0002; 25-OH-D_3_: *P* < 0.0001; 24,25-OH-D_3_: *P* < 0.0001; Figure 2A–C). Although plasma vitamin D_3_ concentration was below the limit of detection for the 400 and 800 IU D_3_/kg treatments, the plasma vitamin D_3_ concentration had a strong positive correlation with 25-OH-D_3_ and 24,25-(OH)_2_-D_3_ concentrations (*r =* 0.95, *P* < 0.0001; *r* = 0.92, *P* < 0.0001; respectively, data not shown). Plasma vitamin D_3_ and 25-OH-D_3_ had similar concentration values when dietary vitamin D_3_ increased, with both metabolites having ∼85 ng/mL at 36,000 IU D_3_/kg treatment. Although plasma 24,25-(OH)_2_-D_3_ concentration was lower than vitamin D_3_ and 25-OH-D_3,_ 24,25-(OH)_2_-D_3_ was affected by dietary treatment, and 24,25-(OH)_2_-D_3_ also exhibited a similar rate of increase relative to dietary treatment like vitamin D_3_ and 25-OH-D_3_. The percentage ratio of 24,25-(OH)_2_-D_3_ to 25-OH-D_3_ increased as dietary vitamin D_3_ concentrations increased and reached an asymptote at the super-dose levels (*P* < 0.0001; Figure 2D). The 24,25-(OH)_2_-D_3_: 25-OH-D_3_ ratio percentage ranged from 8.7% to 20.5%, with all super-dose-fed hens having a ratio of ∼20%.

**FIGURE 3:**
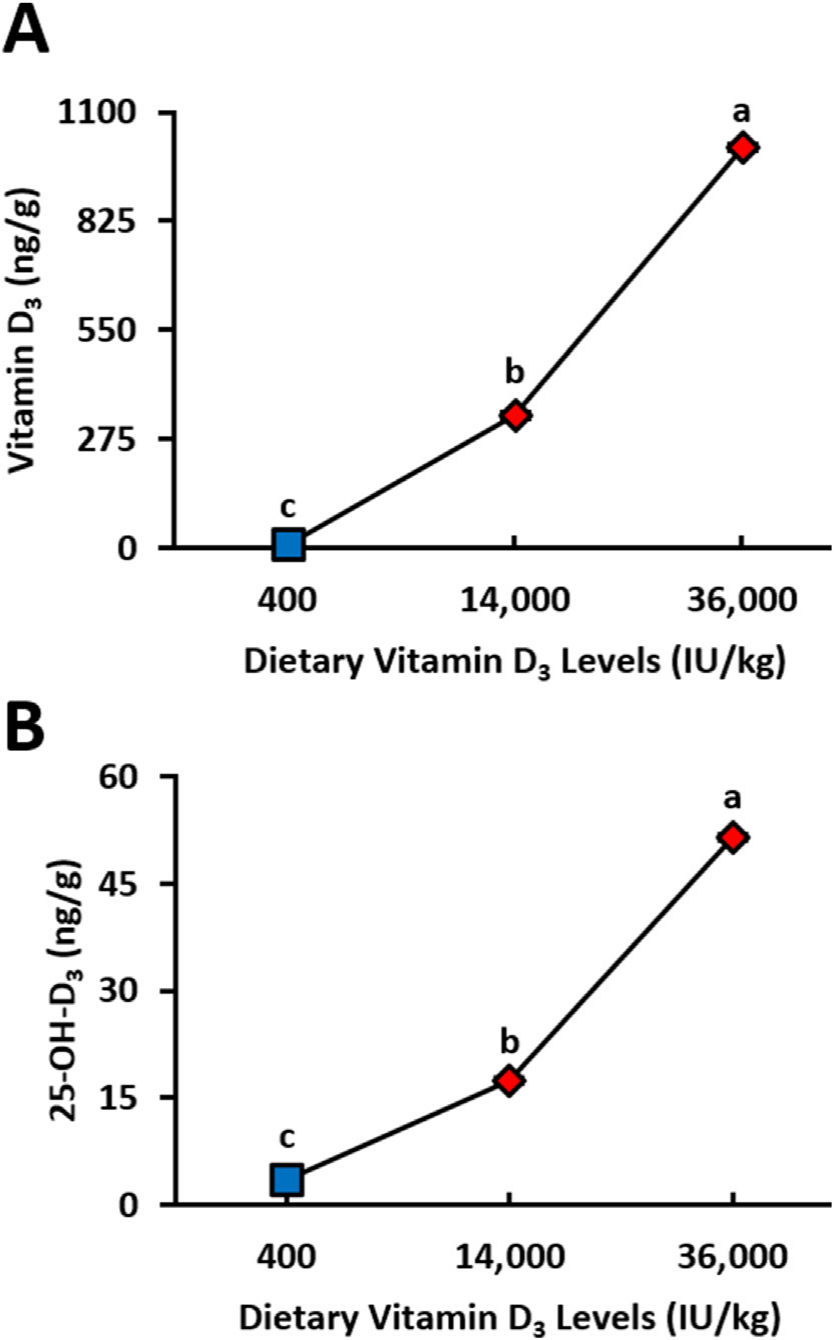
Egg yolk vitamin D_3_ metabolite concentrations from 78-wk Hy-Line Brown laying hens fed different concentrations of dietary vitamin D_3_. (A) Cholecalciferol (vitamin D_3_) (B) 25-hydroxycholecalciferol (25-OH-D_3_). Blue squares denote standard NRC range vitamin D_3_ concentrations (400 IU D_3_/kg) in diet (*n* = 4), and red diamonds denote super-dose concentrations of vitamin D_3_ (14,000 and 36,000 IU D_3_/kg) in diet (*n* = 4). Samples were reported as means ± SEM; however, error bar values were narrow and overlapped by the marker for each sample. Samples with common letters were not significantly different from each other (general linear models, *P* < 0.0001). NRC, National Research Council; SEM, standard error of the mean.

**FIGURE 4:**
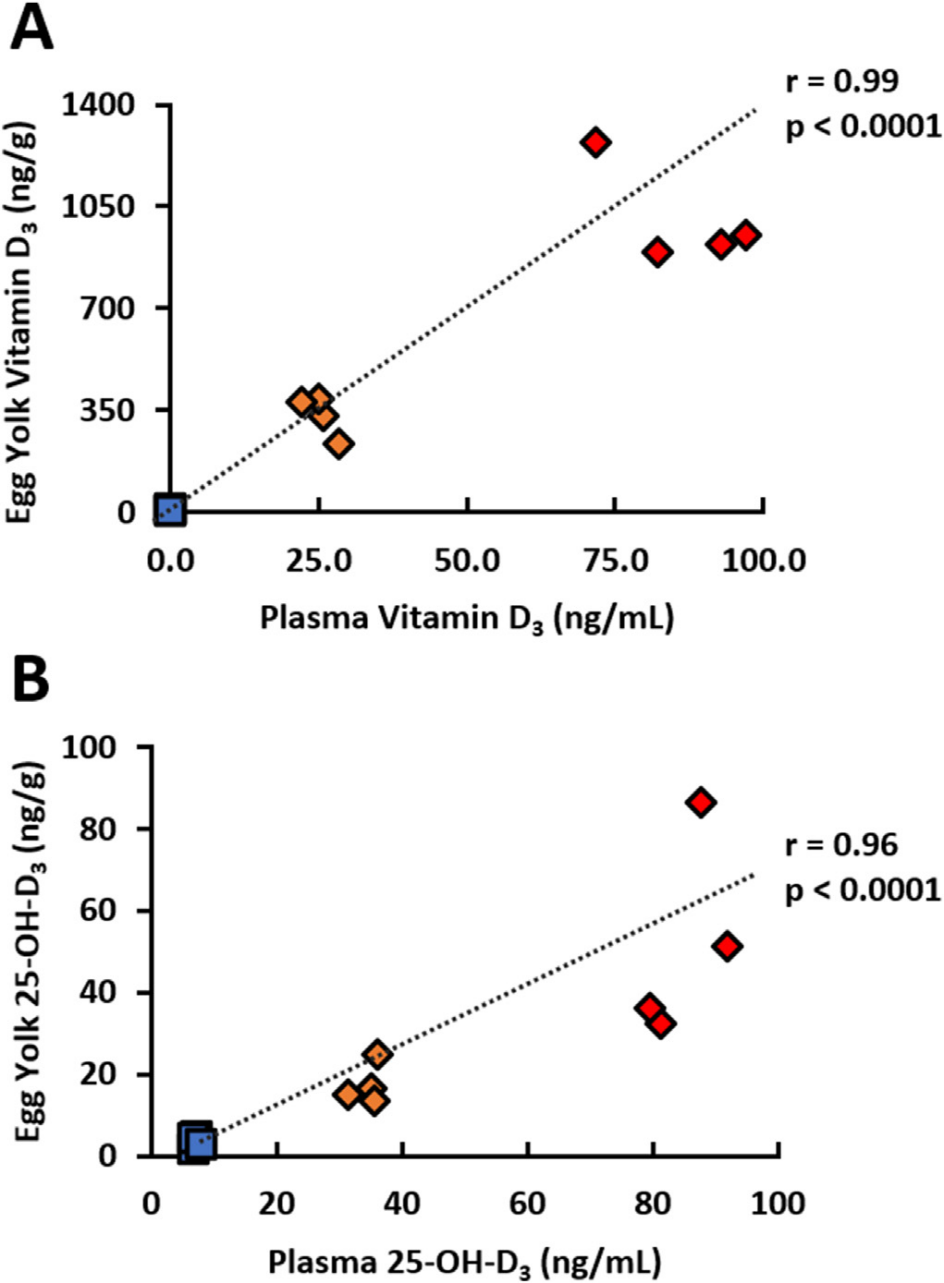
Association between plasma and egg yolk vitamin D_3_ metabolite concentrations from 78-wk Hy-Line Brown laying hens fed different concentrations of dietary vitamin D_3_. (A) Cholecalciferol (vitamin D_3_) (B) 25-hydroxycholecalciferol (25-OH-D_3_). Blue squares denote the standard NRC range vitamin D_3_ concentration (400 IU D_3_/kg, *n* = 4) in diet; orange (14,000 IU D_3_/kg, *n* = 4) and red diamonds (36,000 IU D_3_/kg, *n* = 4) denote super-dose concentrations of vitamin D_3_ in diet (Pearson correlation). NRC, National Research Council.

Egg yolk vitamin D_3_ increased drastically as hens’ dietary vitamin D_3_ intake increased (*P* < 0.0001; Figure 3A and B). Egg yolk 25-OH-D_3_ was also significantly increased in concentration as hens’ dietary vitamin D_3_ increased (*P* < 0.0001), but the rate of increase was much lower compared with egg yolk vitamin D_3_. Egg yolk vitamin D_3_ was strongly and positively correlated with plasma vitamin D_3_ (*r* = 0.99, *P* < 0.0001, Figure 4A). Egg yolk 25-OH-D_3_ also had a strong positive correlation with plasma 25-OH-D_3_ (*r* = 0.96, *P* < 0.0001, Figure 4B).

### Dietary super-doses of vitamin D_3_ intake affected VDR expression and kidney CYP24A1 expression

**FIGURE 5:**
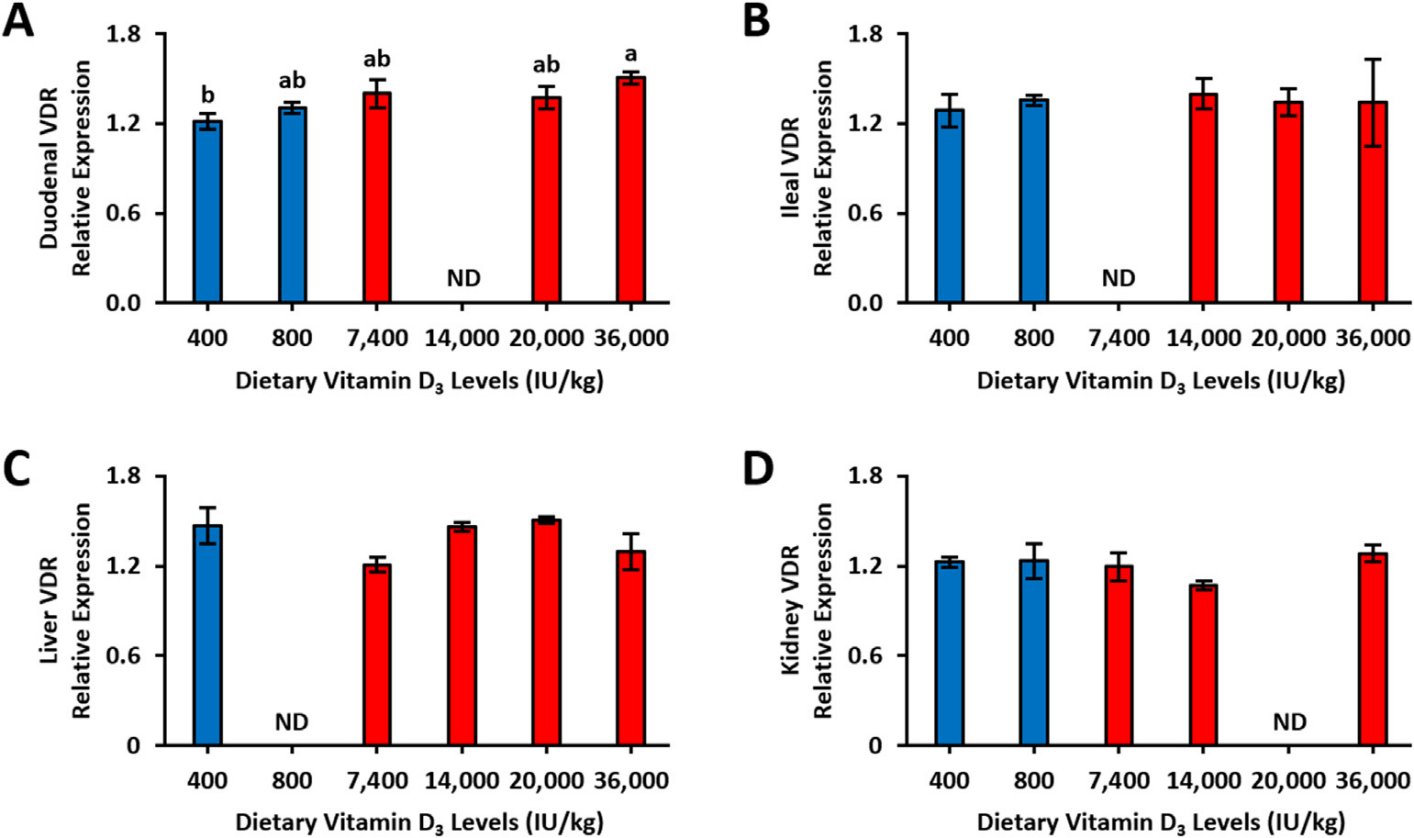
Relative gene expression of vitamin D receptor (VDR) in the duodenum, ileum, liver, and kidney of 78-wk Hy-Line Brown laying hens fed different concentrations of dietary vitamin D_3_. (A) Duodenal VDR (*n* = 2–6) (B) ileal VDR (*n* = 2–5) (C) liver VDR (*n* = 2–4) (D) kidney VDR (*n* = 2–4). Tissues were analyzed using qPCR normalized against glyceraldehyde phosphate dehydrogenase (housekeeping gene) expression. Blue bars denote standard NRC range vitamin D_3_ concentrations in the diet, and red bars denote super-dose concentrations of vitamin D_3_ in the diet. All samples were analyzed in triplicates and reported as means ± SEM. ND = not detected or below limit of detection. Bars with common letters were not significantly different from each other (general linear models, *P* < 0.05). NRC, National Research Council; qPCR, quantitative polymerase chain reaction; SEM, standard error of the mean.

Considering VDR is a ligand-activated transcription factor responsible for exerting vitamin D’s physiologic effects [22], we measured VDR expression in multiple tissues to determine if dietary vitamin D_3_ concentrations would affect VDR expression. Hens fed higher concentrations of dietary vitamin D_3_ had upregulated duodenal VDR expression (*P* = 0.036; Figure 5A). There was no dietary effect on VDR expression from the ileum, liver, and kidney (*P* = 0.96, 0.17, 0.32, respectively; Figure 5B–D). We also examined if vitamin D_3_ super-dosages would affect the gene expression of vitamin D hydroxylase enzymes in the kidney. Unexpectedly, kidney 24-OHase expression was lower in hens-fed diets with super-dose concentrations of vitamin D_3_ (*P* = 0.0006, Supplementary Figure 2A). No differences were observed with kidney 1α-OHase expression (*P* = 0.81, Supplementary Figure 2B).

## Discussion

**FIGURE 6:**
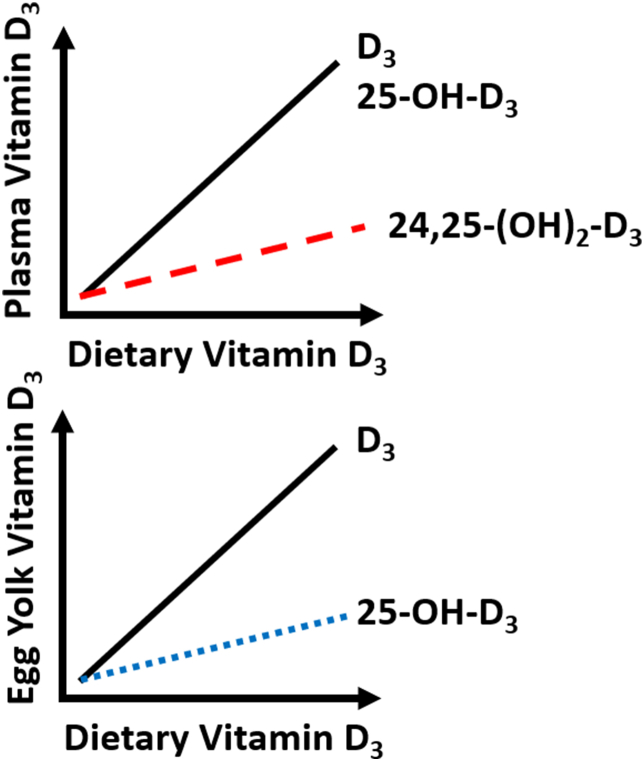
Dietary super-doses of vitamin D_3_ fed to aged laying hens cause drastic increases in plasma and egg yolk vitamin D_3_ metabolites. Egg yolk vitamin D_3_ concentrations were strongly correlated to the dietary concentrations of vitamin D_3_ fed to the hens. Egg yolk 25-hydroxycholecalciferol (25-OH-D_3_) concentrations were also dependent on dietary vitamin D_3_; however, 25-OH-D_3_ increased at a lower rate. For plasma vitamin D_3_ metabolites, vitamin D_3_ and 25-OH-D_3_ concentrations increased relative to dietary vitamin D_3_ concentrations fed to the hens. Plasma 24,25-dihydroxycholecalciferol [24,25-(OH)_2_-D_3_] concentrations were also dependent on dietary vitamin D_3_, but the rate of increase was lower.

Our results suggest that dietary super-doses of vitamin D_3_ greatly increased plasma and egg yolk D_3_ concentrations. Increased plasma vitamin D_3_ indicates these hens absorbed vitamin D_3_ from their diets. Although the inactive vitamin D_3_ metabolite, 24,25-(OH)_2_-D_3_, had a lower measured value than vitamin D_3_ and 25-OH-D_3_, its slope and rate of increase had the same rate of increase. Increasing plasma 24,25-(OH)_2_-D_3_ concentrations highlights that these hens were likely trying to reduce their circulating vitamin D_3_ concentrations (Figure 6). In addition, egg yolk vitamin D_3_ drastically increased, whereas yolk 25-OH-D_3_ had a smaller rate of increase. Altogether, there is a strong association between dietary vitamin D_3_ concentrations and plasma and egg yolk vitamin D_3_ metabolite concentrations.

Although a laying hen’s physiologic status affects egg quality [23], high concentrations of dietary vitamin D_3_ have been shown to reduce egg quality but not affect egg production [24]. Mattila et al. [9] and Wen et al. [10] reported that laying hens fed diets with greater concentrations of dietary vitamin D_3_ throughout their production cycle increased egg yolk vitamin D_3_ content. The egg production and egg yolk vitamin D_3_ data in our study were similar to the 2 aforementioned studies, even though the hens in our study were older. Signs of vitamin D toxicity in laying hens are reduction in egg production and food consumption [12]. However, the hens in our study did not show any drastic differences with either of those which suggests they were in no danger of vitamin D toxicity. A novel finding we observed was that vitamin D_3_ was deposited more readily into the yolk compared with 25-OH-D_3_. One possibility is that excess circulating 25-OH-D_3_ was transferred to the egg yolk as a way to lower circulating 25-OH-D_3_ concentrations. Vitamin D_3_ is readily converted to 25-OH-D_3_ in the liver by the 25-hydroxylase, whereas CYP27C1 and CYP24A1 are tightly controlled by parathyroid hormone and fibroblast growth factor 23 [25,26]. Supplementing laying hen diets with 25-OH-D_3_ increased egg yolk 25-OH-D_3_ and reduced egg yolk vitamin D_3_ [27]. It seems likely that the vitamin D_3_ metabolite composition in egg yolk is influenced by whatever dietary vitamin D_3_ isoform is fed to the laying hens.

The biologic significance of 24,25-(OH)_2_-D_3_ is to reduce 25-OH-D_3_’s plasma concentration [28]. A recent study involving laying hens showed how plasma 24,25-(OH)_2_-D_3_ did not change over time after an egg was laid [29]. In our study, the hens’ plasma 24,25-(OH)_2_-D_3_ increased relative to dietary vitamin D_3_ concentrations. However, unlike plasma D_3_ and 25-OH-D_3_, the rate of increase with plasma 24,25-(OH)_2_-D_3_ was miniscule. The rate of increase for 24,25-(OH)_2_-D_3_ relative to D_3_ and 25-OH-D_3_ at super-dosage level off at 20%, highlighting a possible asymptotic relationship. The asymptote suggests that 24-hydroxylation activity hit its maximal limit. It is not clear if *VDR* expression is associated with plasma 24,25-(OH)_2_-D_3_ concentrations or 24-hydroxylation activity because there was little difference in *VDR* expression across multiple tissues in this study.

Our study has several strengths that highlight its impact on advancing nutritional knowledge. A significant strength of our study is plasma and egg yolk vitamin D_3_ metabolite concentration ranges across treatment groups. This illustrates the experimental design captured a broad range of dietary vitamin D_3_ supplementation concentration effects on plasma vitamin D_3_ metabolites that future research studies can focus on a specific range to build off our findings. Our study provides novel observations of laying hen plasma 25-OH-D_3_ concentrations relative to dietary vitamin D_3_ supplementation that can be valuable for the poultry industry to consider with vitamin D_3_ status. The plasma 24,25-(OH)_2_-D_3_ data are the most exciting finding of our study. Further understanding of the asymptotic relationship of the super-dose concentrations with plasma 24,25-(OH)_2_-D_3_ concentrations can open new knowledge about24,25-(OH)_2_-D_3_’s value as a biomarker for vitamin D metabolism. One possibility of 24,25-(OH)_2_-D_3_’s use as a biomarker is determining a circulating level range that can be used as an early warning sign to suggest a person or animal is starting to approach vitamin D toxicity.

There were a few limitations with this study that were realized when data were collected. We should have investigated the kidney histopathology of these hens because soft-tissue calcification or renal kidney failure could result from the hens reaching vitamin D toxicity [30,31]. However, Mattila et al. [9] did not observe any pathologic issues in kidneys from 67-wk-old hens fed 15,000 IU D_3_/kg of feed. The smaller sample sizes and missing treatment groups from the qPCR results were because some tissue samples would not yield RNA for cDNA synthesis, even after multiple extraction attempts. All tissue samples, except for plasma, were temporarily stored in a 7°C cold room until freezer space was available, which could have caused a reduction in RNA quality. It is important to note that the CYP27C1 gene may not encode CYP27C1 [32] (Table 2 footnote). We also stored the egg yolk in a refrigerator for about a year before the yolk was freeze-dried; however, our findings are similar to Wen et al. [10] to hint toward minimal vitamin D_3_ degradation (Table 3). This could indicate how stable vitamin D_3_ is when it is stored in cold, dark conditions.

**TABLE 3:** Comparison of egg yolk vitamin D_3_ from eggs laid by hens fed different dietary concentrations of vitamin D_3_ in this study compared with Wen et al. 2019 study [10]

This study^1^	Wen et al. 2019 [10]^2^
IU/egg (dietary treatment IU D_3_/kg)	
5.13 (400)	12.6 (1681)
199.7 (14,000)	214.3 (18,348)
605.9 (36,000)	435.5 (35,014)

1 Values were calculated by converting the egg yolk vitamin D_3_ concentration (ng/g) to IU and multiplying by 15 g (the amount used for LC-MS/MS).

2 These values were reported in Figure 1 for [10].

Our study indicates that feeding super-doses of dietary vitamin D_3_ to aged laying hens increases their plasma and egg yolk vitamin D_3_. Importantly, there is a possible metabolic limit of 24-hydroxylation to remove excess circulating vitamin D_3_. Investigating 24-hydroxylation mechanisms will be important to understanding vitamin D_3_ supplementation impacts in geriatric animals for improving bone health and vitamin D metabolism in older humans.

## Author contributions

The authors’ responsibilities were as follows – MFW, KAL: designed research; MFW, DDH, NCT, KAL: conducted research; MFW, KAL: analyzed data; MFW, KAL: wrote manuscript; MFW, KAL: prepared experimental diets for study; MFW, PMP: prepared samples for ashing; MFW, DDH: prepared and shipped plasma and egg yolk samples to Heartland Assays; MFW, KAL: had primary responsibility for final content; and all authors: read and approved the final manuscript.

## Conflict of interest

The authors report no conflicts of interest.

## Funding

This research was supported by the United States Poultry and Egg Association (KAL) and the United States Poultry and Egg Association had no involvement or restrictions regarding publication.

## Data availability

Data described in the current study will be made available from the corresponding author upon request.
